# Intrusion Detection System CAN-Bus In-Vehicle Networks Based on the Statistical Characteristics of Attacks

**DOI:** 10.3390/s23073554

**Published:** 2023-03-28

**Authors:** Junaid Khan, Dae-Woon Lim, Young-Sik Kim

**Affiliations:** 1 Department of Information and Communication Engineering, Dongguk University, Seoul 04620, Republic of Korea; 2 Department of Information and Communication Engineering, Chosun University, Gwangju 61452, Republic of Korea

**Keywords:** intrusion detection, anomaly detection, DoS attack, fuzzy attack, automotive IDS, in-vehicle networks, cyber security, controller area networks

## Abstract

For in-vehicle network communication, the controller area network (CAN) broadcasts to all connected nodes without address validation. Therefore, it is highly vulnerable to all sorts of attack scenarios. This research proposes a novel intrusion detection system (IDS) for CAN to identify in-vehicle network anomalies. The statistical characteristics of attacks provide valuable information about the inherent intrusion patterns and behaviors. We employed two real-world attack scenarios from publicly available datasets to record a real-time response against intrusions with increased precision for in-vehicle network environments. Our proposed IDS can exploit malicious patterns by calculating thresholds and using the statistical properties of attacks, making attack detection more efficient. The optimized threshold value is calculated using brute-force optimization for various window sizes to minimize the total error. The reference values of normality require a few legitimate data frames for effective intrusion detection. The experimental findings validate that our suggested method can efficiently detect fuzzy, merge, and denial-of-service (DoS) attacks with low false-positive rates. It is also demonstrated that the total error decreases with an increasing attack rate for varying window sizes. The results indicate that our proposed IDS minimizes the misclassification rate and is hence better suited for in-vehicle networks.

## 1. Introduction

**Figure 1:**
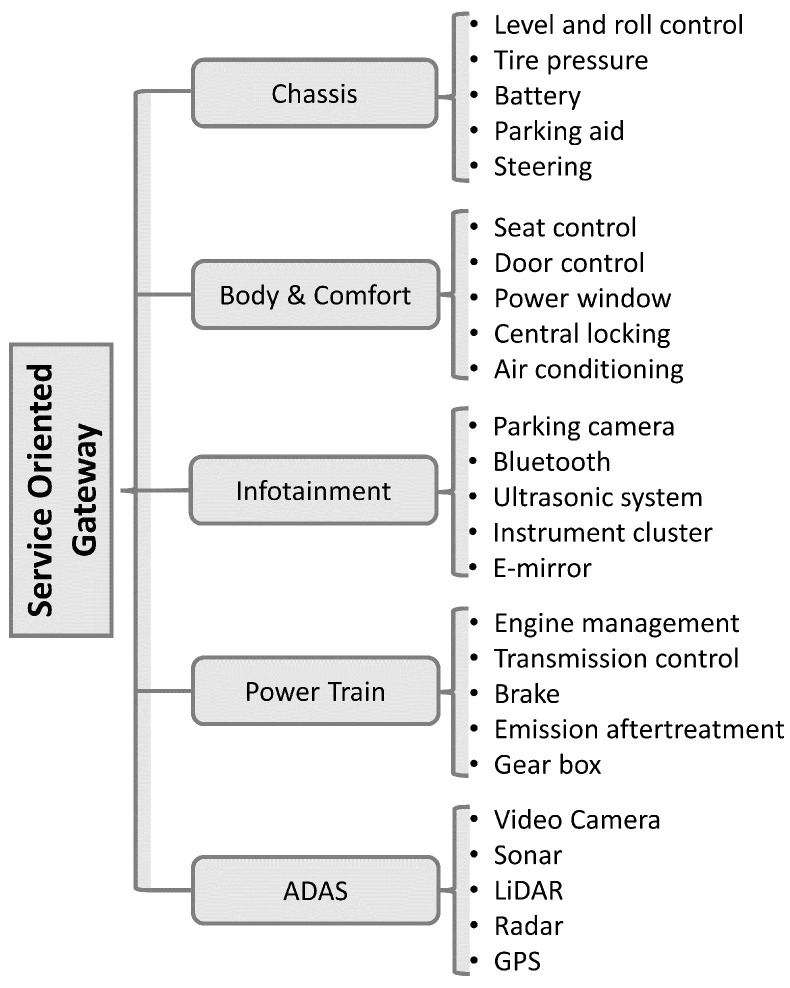
Vehicle domain architecture.

As the automotive industry is rapidly evolving to accommodate industry-driven mechanical and communication technologies, there is a constant need for calibrated actuators, advanced sensors, and high-grade electronic control units (ECUs). The typical structure of a vehicle’s domain architecture is depicted in Figure 1 and is segmented to support the powertrain, chassis, body, and driver assistance tasks connected to many subsystems. The complexity of such subsystems used in vehicles increases with each hardware addition [1]. To comply with industry requirements, the controller area network (CAN) bus is the classic in-vehicle automotive network, allowing vehicles to reduce wiring complications and reap the benefits of design simplification. Since the CAN network is primarily responsible for real-time communication between the connected ECUs and in-vehicle automotive network, data must accurately and reliably flow with extremely low latency [2]. Such a data-intensive application is inherently subject to malicious attacks; therefore, the automotive-based applications of the CAN bus require cutting-edge intrusion detection systems (IDS) with high accuracy and detection rates.

One way to protect the CAN bus communication for the in-vehicle automotive network is by incorporating security elements such as a reliable IDS capable of detecting various attack scenarios. Extensive research has been carried out on CAN bus IDS for in-vehicle automotive networks [3,4,5]. While the effects of CAN bus vulnerabilities have necessitated an upgrade of security requirements for in-vehicle networks over the last decade, an integrated IDS has proven to have significantly enhanced the security features of automotive applications [6,7].

Numerous intrusion detection methodologies have been developed to prevent emerging attacks targeting in-vehicle networks, but they also have certain limitations. For instance, several machine learning (ML)-based techniques have been established for anomaly detection in the CAN network, but the initial model parameter training usually requires a lot of computing power and expensive hardware, making them useless for the automotive industry as a whole [8,9,10]. Message authentication code (MAC) protocols can secure in-vehicle networks, but CAN-bus bandwidth limitation hinders the advancement in this area, which also renders most cryptographic algorithms impractical [11,12,13]. MAC-supported protocols also require altering the mechanisms by which the firmware operates or the way ECUs talk to each other, making them unfavorable for CAN bus security. The potential of parametric-based intrusion detection approaches has also been discussed in the literature. According to [14,15,16,17], the study of frequency-based IDS and the resulting outcome is an essential aspect of in-vehicle CAN bus security. However, frequency-based IDS that calculate the inter-packet timing of CAN bus frames or learn periodicity patterns for anomaly detection have significant drawbacks, such as a few advanced attacks that make these IDS ineffective by gradually changing the periodicity or content of data frames. Windowing and thresholds are used to find intrusions in CAN bus traffic. However, most IDS have not selected the optimal values for these parameters for intrusion detection. Motivated by this, we investigated into obtaining optimally tuned IDS parameters in order to maximize IDS detection and address the limitations in [18,19,20,21,22,23]. We focus on a system model in which ECUs take measurements in real-time and send the results to a service-oriented gateway via different types of CAN buses.

In this manuscript, we present an innovative parametric method to develop CAN network IDS, where we investigate both experimental relationships and numerical analysis for the attack ratio, average, and standard deviation of CAN bus data. We obtained the lowest possible error rates by optimizing the threshold values for various window sizes. We evaluated the error rate for denial-of-service (DoS) and fuzzy attacks using a variety of performance metrics. Fuzzy attacks introduce arbitrary dataframes into the CAN network, generating complicated network traffic [24]. In the real world, attackers combine multiple attacks; to circumvent this situation, we generated a merge attack by combining DoS and a fuzzy attack and conducted a thorough analysis. This study makes use of two datasets obtained from real-world CAN-bus in-vehicle networks to determine whether or not our proposed IDS is credible. Optimal threshold values are obtained using a brute-force optimization algorithm to identify anomalies in the in-vehicle network.

To the best of our knowledge, no prior research has suggested an IDS based on statistical characteristics of attacks that optimizes threshold values and uses optimal window sizes to minimize detection error rates using a sliding window approach for the CAN-bus in-vehicle network. Our major contributions to this manuscript are listed below:

1. A novel IDS based on the statistical characteristics of attacks is proposed to identify CAN-bus anomalies for the in-vehicle network. The IDS is equipped with a sliding window-based intrusion detection function.
2. A merge attack was developed by combining DoS and fuzzy attack to evaluate proposed IDS against real-life attack scenario [25]. Experimental results demonstrate that the suggested technique can efficiently detect fuzzy, merge and DoS attacks with a high degree of detection accuracy.
3. Optimize threshold values are obtained using the brute-force optimization method and variation in window size is examined thoroughly to obtain minimum misclassification rate.
4. The proposed methodology is fine-tuned by adjusting parameters to obtain optimal results and conducting a thorough analysis using a variety of performance metrics.
5. The credibility of the proposed IDS is tested against two real datasets, i.e., the car-hacking dataset [26] and the survival analysis dataset [27].

The remaining sections of this manuscript are organized as follows: Section 2 summarizes previous research on anomaly detection for the in-vehicle automotive network. Section 3 discusses the precise design details of our proposed anomaly detection system, whereas Section 4 discusses the experimental outcomes. In Section 5, we discuss the limitations of our research and our future objectives. Finally, in Section 6, we summarize our findings, and Table 1 describes the notations used throughout this study.

## 2. Related Work

This section discusses the CAN bus protocol, CAN-FD, various vulnerabilities that affect in-vehicle CAN bus networks, the methodologies used to develop vehicular IDS, and the practical limitations of these systems.

### 2.1. CAN Bus Protocol

**Figure 2:**
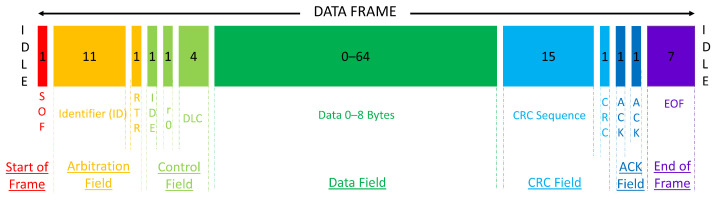
CAN-bus data frame format.

One of the most prevalent multi-master serial communication buses, the CAN bus, can support baud rates up to 1 Mbit/s. At first, it was intended for the automotive industry only, but it is now being used in most industrial applications. The CAN bus is equipped with crucial attributes such as self-diagnosis, which allows for identifying component failures. The CAN bus mechanism has been fine-tuned to provide a robust response to safety-critical systems when necessary. The CAN bus includes error correction capabilities as part of its effort to ensure reliable communication between ECUs [28]. Despite these significant advantages, it falls short of some fundamental requirements for secure data communication between critical subsystems connected to the in-vehicle networking system. As illustrated in Figure 2, the CAN bus inherent design lacks message authentication, and it also does not support point-to-point data transmission between nodes.

All of the ECUs can take advantage of the network’s unsegmented nature to broadcast messages and communicate directly with safety-critical subsystems, which is advantageous for all of them. Because CAN bus traffic is not encrypted, it can be easily sniffed, spoofed, modified, and replayed to inject various types of attacks, such as DoS, fuzzy, and personification attacks [29]. These vulnerabilities necessitate the use of some robust intrusion detection systems that can be added to complex intrusion detection systems and perform quick initial diagnostics. The communication technology used in vehicle networks has advanced significantly in recent years. CAN with Flexible Data Rate (CAN-FD) is introduced to be used in the next generation of automotive systems [30]. Although the CAN-FD supports advanced features, i.e., a communication data rate greater than 1 Mbit/s and an increased payload size of up to 8 Mbit/s [31], security issues can still prevail in the practical use of the CAN-FD [32].

### 2.2. Methods for In-Vehicle Intrusion Detection

Attackers may acquire unauthorized control of connected vehicles by inserting malicious data frames into the in-vehicle networks, most notably the CAN bus. Numerous intrusion detection approaches have been developed to guard against CAN message eavesdropping, and Table 2 reviews the common limitations and specific contributions of relevant studies. According to their detection scope, intrusion detection algorithms may be classified as fingerprint-based, parametric-based, entropy-based, or deep learning-based (DL-based) methods.

#### 2.2.1. Fingerprint-Based Methods

Groundbreaking work by Cho et al. demonstrated that fingerprints of hardware could be used to model the clock’s behavior [22]. The hardware generates unique fingerprint information because of the inherent physical properties, and their IDS uses the cumulative sum method on the fingerprint data collected to detect any possible abnormal behavior of the in-vehicle network. The algorithm calculates a clock offset based on message periodicity, limiting its ability to detect intrusions for aperiodic information. Later in [33], it was also observed that a voltage-based attacker identifier scheme called Viden could be built using voltage measurements to identify attackers. Voltage fingerprints of ECUs using transmitter voltages to create voltage profiles. However, voltage profiles for ECUs are required during the manufacturing stage and are updated through voltage profile adjustments for accurate detection. Researchers in [34] observed that the extra wires needed by voltage-based IDS might introduce various voltage-based attacks into the CAN bus. Moreover, due to hardware failure in their IDS, fuse and circuit breakers must be manually replaced. A similar method was used by Li et al. [35], and exploited temperature variation impact on the voltage characteristics to obtain hardware fingerprints. They showed that the temperature-varied voltage fingerprinting scheme solution is optimal compared to other fingerprint-based IDS. Nevertheless, real-time signal measurement can be a difficult job in a constrained environment and may hinder the implementation of the proposed methods.

#### 2.2.2. Parametric-Based Method

Taylor et al. [14] detected malicious messages using a Hamming distance between data frames and interpacket timing-based statistics features and indicated that a significant amount of data was required to achieve a low false-positive rate. Song et al. [36] suggested a frequency-based, lightweight IDS for the CAN bus to determine whether the vehicle has been attacked by data injection. The system uses the time interval between CAN data frames for anomaly detection. However, it requires more computing power to analyze the CAN message sequence in order to improve the detection accuracy.

#### 2.2.3. Entropy-Based Methods

Muter and Asaj demonstrated the idea of an entropy-based method for CAN-bus network anomaly detection. Their anomaly detection method calculated the ID frequency for in-vehicle network [37]. The limitation of the approach includes the difficulty of recognizing small-scale attacks. Marchetti et al. assessed the usefulness of an entropy-based intrusion detection method for modern vehicles. Based on experimental data, they showed that accurate attack detection could only be obtained if the abnormal data size was large [38]. Wu et al., used the entropy-based method and enhanced the detection accuracy for in-vehicle automotive network attacks while maintaining a low response time. They also optimized the size of sliding window [39]. However, calculating the intrusion detection threshold in entropy-based IDS is challenging.

#### 2.2.4. Deep Learning-Based Methods

Seo et al. suggested a generative adversarial network (GAN)-based IDS for in-vehicle automotive network using a deep learning method. The GAN-based intrusion detection system (GIDS) was trained on a fake random dataset to detect attacks in real CAN bus data [40]. Moreover, GIDS is incapable of detecting malicious or component-failure data. Later, in [41], CANnolo was proposed to use long short-term memory (LSTM) autoencoders to identify intrusions in CAN bus data. CANnolo generated a model using normal data and detected intrusion based on the difference between reconstructed and real CAN bus data, but requires complex computation for in-vehicle environment. Amato et al., in their latest work, suggested a method based on deep learning to detect attacks on CAN-bus data frames [42]. Their work aimed to detect malicious attacks based on the human behavior of attackers. The algorithm applies multilayer perceptrons (MLP) to train the algorithm. However, the lack of ensemble learning limits the performance of the model.

## 3. Proposed Methodology

This section is divided into two subsections: proposed intrusion detection method and performance metrics.

### 3.1. Proposed Intrusion Detection Method

This research aimed to propose a novel intrusion detection system (IDS) to identify anomalies, particularly in-vehicle networks. This methodology implements a fixed sliding window-based intrusion detection function. The average of averages and the average of standard deviations were used to calculate the reference values of normality and the threshold values. An abnormality condition for *i*-th window vector is detected by comparing the mean $\mu^{i}$ and standard deviation $\sigma^{i}$ of *i*-th window with $\mu_{a}$, $\mu_{s}$, $\sigma_{a}$, and $\sigma_{s}$, respectively. The abnormality in the *i*-th window vector **W**${}_{N}^{i}$ is determined by value of intrusion detection function, $f(\mu^{i},\sigma^{i})=0$ indicates that the *i*-th window vector **W**${}_{N}^{i}$ is a normal window vector while $f(\mu^{i},\sigma^{i})=1$ represents the abnormal state for the in-vehicle network data. Let **D** $=[d_{1}\;d_{2},\cdots ,d_{k},\cdots ,d_{K}]$ be the identifier vector where *K* is the total number of data frames and $d_{k}$ is the identifier of the *k*-th data frame for 1≤k≤K. Since the size of an identifier in the data frame is 11 bits, there are $Q=2^{11}=2048$ identifiers in the data frames. Thus, $d_{k}$ is given by $0\leq d_{k}<Q$. Let the *i*-th window vector **W**${}_{N}^{i}$ be given by **W**${}_{N}^{i}=\left[d_{(i-1)N+1},d_{(i-1)N+2},\cdots ,d_{(i)N}\right]$ for 1≤i≤K/N, where *N* is the window size and it is assumed that *K* is a multiple of *N*. Let **Y**${}^{i}=\left[y_{1}^{i},y_{2}^{i},y_{3}^{i},\cdots ,y_{Q}^{i}\right]$ be the *i*-th window’s frequency vector where $y_{q}^{i}$ is the frequency of the *q*-th identifier in **W**${}_{N}^{i}$ for 1≤q≤Q. Then, we have $\sum_{q=1}^{Q}y_{q}^{i}=N$ as the sum of all the frequency identifiers. Let $I^{i}=\left\{j|y_{j}^{i}>0,1\leq j\leq Q\right\}$ be the index set of identifiers which have non-zero frequency in the *i*-th window, where $|I^{i}|$ denotes the number of elements in $I^{i}$. Let $\mu^{i}$ and $\sigma^{i}$ be the average and standard deviation of non-zero elements in **Y**${}^{i}$ as follows:

(1) $$\mu^{i}=\frac{1}{|I^{i}|}\sum_{q\in I^{i}}y_{q}^{i}=\frac{N}{|I^{i}|}$$

(2) $$\sigma^{i}=\sqrt{\frac{1}{|I^{i}|}\sum_{q\in I^{i}}{\left(y_{q}^{i}-\mu^{i}\right)}^{2}}$$

#### 3.1.1. Reference Values of Normality

Let us assume that the first *L* window vectors **W**${}_{N}^{i}$, 1≤i≤L contain normal CAN-bus data frames with no attacks. Then, they are used for calculating the reference values of normality. Let $\mu_{a}$ and $\sigma_{a}$ be the average and standard deviation of the averages of first *L* window frequency vectors **Y**${}^{1}$, **Y**${}^{2}$, *…*, **Y**${}^{L}$:

(3) $$\mu_{a}=\frac{1}{L}\sum_{l=1}^{L}\mu^{l}$$

(4) $$\sigma_{a}=\sqrt{\frac{1}{L}\sum_{l=1}^{L}{\left(\mu^{l}-\mu_{a}\right)}^{2}},$$

respectively. Let $\mu_{s}$ and $\sigma_{s}$ be the average and standard deviation of the standard deviations of **Y**${}^{1}$, **Y**${}^{2}$, *…*, **Y**${}^{L}$:

(5) $$\mu_{s}=\frac{1}{L}\sum_{l=1}^{L}\sigma^{l}$$

(6) $$\sigma_{s}=\sqrt{\frac{1}{L}\sum_{l=1}^{L}{\left(\mu^{l}-\mu_{s}\right)}^{2}}$$

#### 3.1.2. Abnormality Identification

Abnormality in *i*-th window vector **W**${}_{N}^{i}$ is decided by computing the intrusion detection function $f(\mu^{i},\sigma^{i})$. The outcome of intrusion detection function $f(\mu^{i},\sigma^{i})$ depends on values of $\mu^{i}$, $\sigma^{i}$, $\mu_{a}$, $\sigma_{a}$, $\mu_{s}$, and $\sigma_{s}$ and is given as

(7) $$f\left(\mu^{i},\sigma^{i}\right)=\{\begin{array}{cccc} 0,\;\; & \frac{|\mu^{i}-\mu_{a}|}{\sigma_{a}}\leq \gamma_{1} & and & \frac{|\sigma^{i}-\mu_{s}|}{\sigma_{s}}\leq \gamma_{2}\\ 1,\;\; & otherwise, \end{array}$$

where $\gamma_{1}$ and $\gamma_{2}$ are threshold values. Then, $f(\mu^{i},\sigma^{i})=0$ indicates that the *i*-th window vector is normal while $f(\mu^{i},\sigma^{i})=1$ represents the abnormal state. The attack ratio $\epsilon^{i}$ of the *i*-th window vector **W**${}_{N}^{i}$ is defined as

(8) $$\epsilon^{i}=\frac{M^{i}}{N}$$

where $M^{i}$ is the number of attack frames in **W**${}_{N}^{i}$. Table 1 lists the notations used in this methodology to identify different parameters used.

The algorithm can be summarized in the following steps:

1. Preprocessing
  - Each window vector **W**${}_{N}^{i}$ is extracted from the CAN bus dataset.
  - For each of the *i*-th window vector **W**${}_{N}^{i}$, a frequency vector **Y**${}^{i}=\left[y_{1}^{i},y_{2}^{i},y_{3}^{i},\cdots ,y_{Q}^{i}\right]$ is calculated.
2. Calculation of reference values of normality
  - Using legitimate CAN-bus data frames, *L* frequency vectors **Y**${}^{1}$, **Y**${}^{2}$, *…*, **Y**${}^{L}$ are calculated.
  - Reference values of normality, i.e., average of averages $\mu_{a}$, standard deviation of the averages $\sigma_{a}$, average of standard deviations $\mu_{s}$, and standard deviation of the standard deviations $\sigma_{s}$ are calculated.
3. Intrusion detection
  - For each *i*-th frequency vector **Y**${}^{i}$, the average $\mu^{i}=\frac{1}{|I^{i}|}\sum_{q\in I^{i}}y_{q}^{i}=\frac{N}{|I^{i}|}$ and standard deviation $\sigma^{i}=\sqrt{\frac{1}{|I^{i}|}\sum_{q\in I^{i}}{\left(y_{q}^{i}-\mu^{i}\right)}^{2}}$ are calculated using the index set of identifiers $I^{i}=\left\{j|y_{j}^{i}>0,1\leq j\leq Q\right\}$.
  - $f(\mu^{i},\sigma^{i})$ is used to decide the abnormality of the *i*-th window vector **W**${}_{N}^{i}$ by comparing $\mu^{i}$ and $\sigma^{i}$ with $\mu_{a}$, $\sigma_{a}$, $\mu_{s}$, and $\sigma_{s}$.

### 3.2. Performance Metrics

To evaluate the effectiveness of the suggested method against the attacks described in the previous section, we have calculated the true positive, true negative, false positive, false positive error rate, false negative, false negative error rate, misclassification rate, total error, average error rate, F1-score, recall, precision, and accuracy. Table 3 describes the performance parameters used in this study. Essentially, false negative may occur when *i*-th window vector **W**${}_{N}^{i}$ is malicious, but detector prediction is normal. If a false negative occurs, the number of false negatives is increased by 1 based on the condition $\epsilon^{i}\geq \alpha$ & $f(\mu^{i},\sigma^{i})=0$, where α is the minimum value of attack ratio for 0≤α≤0.02. Conversely, the number of false positives is incremented by 1 when the detector prediction indicates an anomaly for actually normal *i*-th window vector **W**${}_{N}^{i}$ based on condition $\epsilon^{i}=0$ & $f(\mu^{i},\sigma^{i})=1$. A true negative is defined as an event when both the *i*-th window vector **W**${}_{N}^{i}$ and detector prediction are normal. If a true negative occurs, the number of true negatives is increased by 1 based on the condition $\epsilon^{i}=0$ & $f(\mu^{i},\sigma^{i})=0$. Conversely, the number of true positives is incremented by 1 when both the *i*-th window vector **W**${}_{N}^{i}$ and detector prediction are abnormal based on condition $\epsilon^{i}\geq \alpha$ & $f(\mu^{i},\sigma^{i})=1$.

False positive error rate (FPER), or the *fall out* is the fraction between the number of legitimate windows incorrectly identified as abnormal and the total number of actual legitimate windows:

(9) $$FPER=\frac{FP}{FP+TN}$$

False negative error rate (FNER) or the *miss rate* is the fraction between the abnormal windows identified as normal and the total abnormal windows:

(10) $$FNER=\frac{FN}{FN+TP}$$

Total error (TE) is defined as total number of incorrect decisions:

(11) TE=FP+FN

Average error rate (AER) is the rate obtained by averaging the sum of FPER and FNER:

(12) $$AER=\frac{FPER+FNER}{2}$$

Misclassification rate (MR) is described by the total number of errors made during the prediction divided by the total windows:

(13) $$MR=\frac{FP+FN}{FP+TP+FN+TN}$$

Accuracy (ACC) denotes the ratio of accurately classified samples in the whole sample space and can be calculated as follows:

(14) $$ACC=\frac{TP+TN}{TP+FP+FN+TN}$$

Precision (PRC) refers to the proportion of malicious windows correctly classified to the total number of attack instances:

(15) $$PRC=\frac{TP}{TP+FP}$$

Recall (RCL) shows what percentage of all windows that have been identified as being attacked are actually attacked:

(16) $$RCL=\frac{TP}{TP+FN}$$

The F1-score is clearly linked to precision and recall. The F1-score value fluctuates between 1 and 0 and can be viewed as the average of model precision and recall:

(17) $$F1=2\left(\frac{RCL\times PRC}{RCL+PRC}\right)$$

## 4. Experimental Results

This section is divided into seven subsections: dataset description, experiments, and evaluations.

### 4.1. Datasets Description

Two datasets were used in this study to validate the credibility of our proposed IDS. The car hacking dataset was initially published by [26] and the survival analysis dataset was introduced by [27]. The datasets were extracted by categorizing CAN traffic via the OBD-II port of a vehicle while attacks were being performed. In addition to a timestamp and CAN identifier, an 8-bit data field (DATA [0–7]) and the data length code (DLC) are also included in the datasets. We utilized data collected from real vehicles to validate the generality of the proposed intrusion detection method in our paper. The datasets contained data from three different automobile manufacturing companies, namely Hyundai, Kia, and Chevrolet, and were used as a training and testing dataset to effectively design our proposed IDS. Hu et al., in [25] used a multi-attack scenarios for in-vehicle intrusion detection system. We have combined DoS and fuzzy attacks to create a merged attack and created a similar attack scenario as proposed in [25] using the car hacking dataset [26]. The main features of all the datasets used in the experimentation of our study are provided in Table 4.

The DoS and fuzzy attack datasets are publicly available for research purposes. The merged attack dataset is formed by combining the smaller blocks of the original DoS and fuzzy datasets. The merged dataset contains 25,000 data frames alternately combined from each dataset. A total of 3,000,000 data frames are combined to create a merged attack, extracted from DoS and fuzzy attack datasets. A block of 25,000 data frames is copied from the DoS dataset, following another block of 25,000 data frames from the fuzzy attack dataset. In total, 1,500,000 data frames from each dataset are merged to make a new dataset for experimentation.

### 4.2. Attack Ratio vs. Average and Standard Deviation

**Figure 3:**
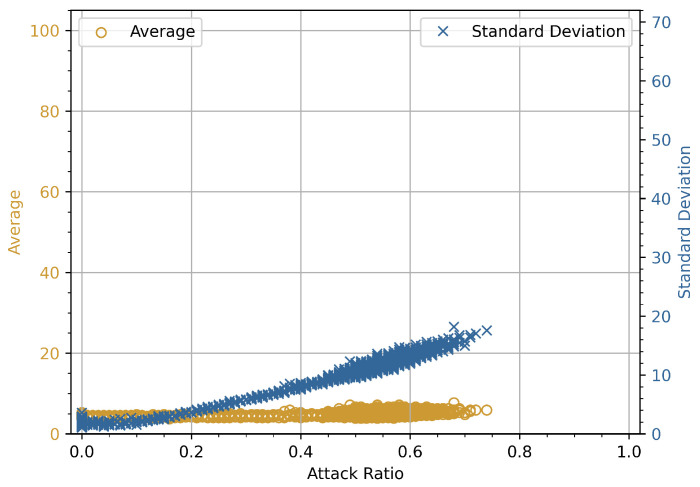
Experimental results of attack ratio vs. average and standard deviation for DoS attack.

**Figure 4:**
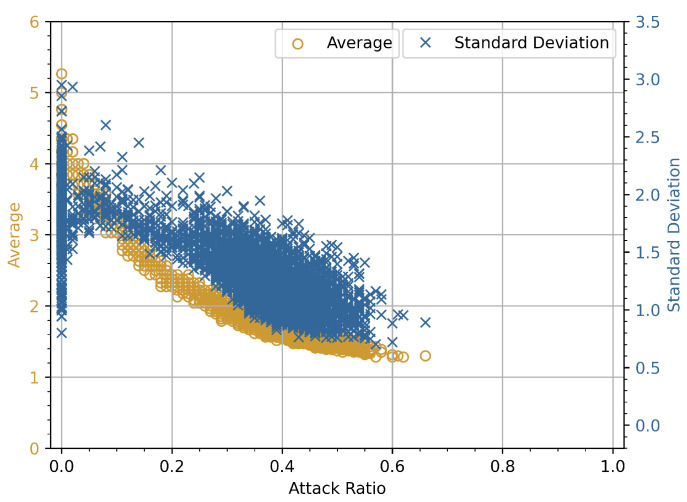
Experimental results of attack ratio vs. average and standard deviation for fuzzy attack.

**Figure 5:**
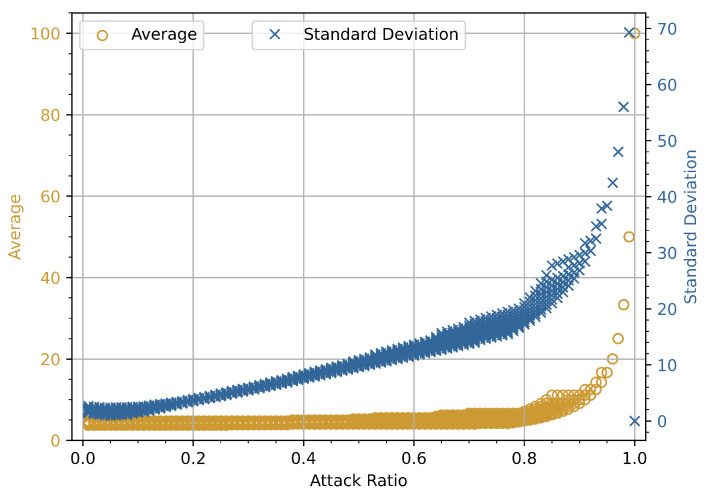
Simulation results of attack ratio vs. average and standard deviation for 100% DoS attack.

**Figure 6:**
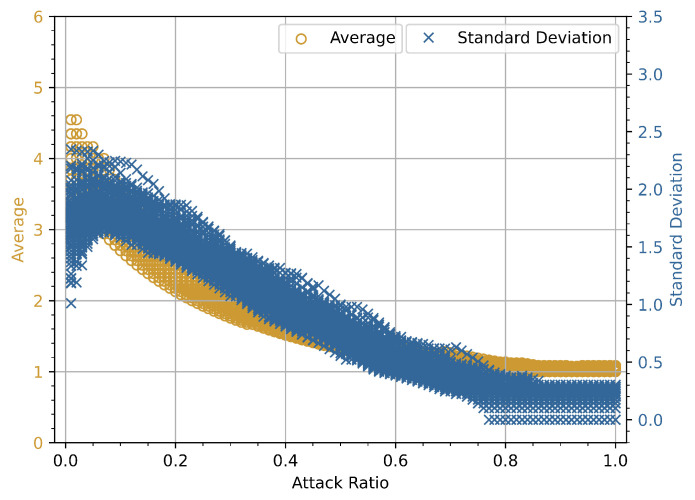
Simulation results of attack ratio vs. average and standard deviation for 100% fuzzy attack.

The experimental relationship and numerical analysis for the attack ratio plotted against the average and standard deviation (SD) for DoS and fuzzy attacks on the x and double-y-axes are presented in the following subsections. The graphs in Figure 3, Figure 4, Figure 5 and Figure 6 show the average and standard deviation for each of *i*-th window vector **W**${}_{N}^{i}$. We used a double *y*-axis graph to verify the relationships between average and SD with various ranges to get a broader view of whether both dependent variables increase or decrease with a change in attack ratio. *N* is set to have 100 identifiers in each of *i*-th window vector **W**${}_{N}^{i}$ as a predetermined value. We are determining the statistical characteristics of attacks in this experiment, so the normality reference values are not measured. In the course of this investigation, *K* = 100,000 individual data frames are extracted from the CAN-bus car hacking dataset. The highest possible value for *i*-th index will be 1000 for this parameter configuration.

#### 4.2.1. Experimental Relationship

The priority order of the CAN frames is critical in CAN bus communication. Assume that the highest-order CAN bus frame is continuously injected into the in-vehicle network. It will result in a DoS attack scenario, and legitimate traffic will be hampered as a result of the priority order. The *x*-axis in Figure 3 represents the attack ratio, while the double *y*-axis denotes the average and SD values of the CAN bus frames, respectively. The average and SD have lower values in the absence of an attack. However, the average and SD values gradually rise under attack conditions as the attack rate increases. In the beginning, the attack rate was around 5–10%, and malicious frames were scarce compared to legitimate CAN bus frames. This is due to the fact that the maliciously inserted CAN bus frames are comparable to the rest of the CAN bus frames. However, as the attack rate went up from 10% to 70%, the malicious CAN bus frames became the majority, with an overall increase in average and SD values for each window. The two curves are slightly different, as shown in Figure 3, and these graphs indicate that the average value increases linearly. However, neither the average nor the standard deviation showed an increasing trend in this experiment. During the course of a fuzzy attack, randomly generated malicious frames are successively injected into the in-vehicle network. Because each ECU connected to the CAN bus accepts all frames during the fuzzy attack, the average and SD values decrease in contrast to the increase in attack rate. This decreasing trend is clearly visible in Figure 4, where the average and SD values of attacked frames decreased as the attack rate increased.

#### 4.2.2. Numerical Analysis

We present a numerical analysis for the above experiment performed for CAN bus traffic. It can be easily observed from Figure 3 and Figure 4 that for a predetermined size window vector **W**${}_{N}^{i}$, a DoS attack increases the average and the standard deviation, and a fuzzy attack decreases them for *N* identifiers, i.e., $d_{(i-1)N+1},d_{(i-1)N+2},\ldots ,d_{(i)N}$. However, if we focus on Figure 3, as the intensity of a DoS attack is increased by injecting continuous data frames, the average value of the window vector **W**${}_{N}^{i}$ increases, while the standard deviation either remains constant or decreases. Similarly, in Figure 4, as the rate of injections containing fuzzy frames increased, the average and standard deviation values showed a decreasing trend. We cannot deduce a statement based on these observations because the maximum number of possibly attacked frames in the available car hacking dataset is less than 80% as seen in Figure 3 and Figure 4. To create an extreme scenario, i.e, a 100% DoS or fuzzy attack condition in which the attack ratio $\epsilon^{i}$ is equal to 1, we consider the following. The average will attain maximum value and the standard deviation will become zero, since there will be only one identifier $d_{i}$ in the *i*-th window vector **W**${}_{N}^{i}$, which is used to inject the attack. Considering this extreme case and the simulation results shown in Figure 3 and Figure 4, we cannot verify the conjecture with the dataset provided by Korea University because the attack ratio remains below 80% throughout the whole dataset.

To overcome this hurdle, we used the existing car hacking dataset to create a 100% attack scenario. Malicious data frames from each attack dataset are used to obtain an attack rate $\epsilon^{i}$ be 0.8 or higher and performed the simulations as shown in Figure 5 and Figure 6. Fixed size window vectors **W**${}_{N}^{i}$ containing 100 legitimate data frames (*N* = 100) were selected from each dataset. One data frame in the window vector at a time is replaced by an attack data frame. After replacing the legitimate data frame with a malicious data frame, the average, standard deviation, and attack ratio of each window vector are calculated. This process was repeated for 1000 window vectors containing legitimate data frames.

The results in Figure 5 show that increasing the DoS attack to 100% can achieve a maximum average value and a minimum standard deviation value. Only CAN-bus data frames with the highest priority were injected into the malicious window during the DoS attack. Similarly, Figure 6 depicts the effect of a 100% fuzzy attack; increasing the attack rate reduces the average and standard deviation values linearly. For the fuzzy attack, the malicious window contains various data frames that reduce the average value during the fuzzy attack period.

### 4.3. Misclassification Rate vs. Attack Ratio

In the following experiment, different fixed-size windows are used, i.e., *N* can have a value of 50, 100, 200, 500, and 1000 in each of *i*-th window vector **W**${}_{N}^{i}$. For this experiment, K= 2,500,000 data frames are analyzed using the car hacking dataset. For each value of *N*, the *i*-th index has a maximum value of 50,000, 25,000, 12,500, 5000, and 2500. The reference values of normality are calculated using only legitimate CAN bus data frames from DoS and fuzzy attack datasets, respectively. A total *L* = 100 window vectors are analyzed to obtain the reference values of normality. For each of *N*, the reference values of normality are computed accordingly by changing the window vector **W**${}_{N}^{i}$. The $\epsilon^{i}$ is divided into ten smaller blocks, each of length 0.1 between 0 and 1.0. Misclassification is calculated for each smaller block. In a binary classification problem, the MR is proportional to the rate at which the fraction of predicted values is incorrect. The MR is defined as the sum of all prediction errors divided by the total number of instances. The following experiment investigates the MR for a predetermined window vector **W**${}_{N}^{i}$ against DoS and fuzzy attacks. The following experiment will look at how the MR varies for different window sizes against DoS and fuzzy attack datasets. The parameters and criteria for intrusion detection are almost identical to those used in the previous experiment; the only major variable is **W**${}_{N}^{i}$, which represents the predetermined window size. For each window used, the reference values of normality are computed. The number of CAN frames used to assess the intrusion using the intrusion detection function $f(\mu^{i},\sigma^{i})$ is specified by the window vector **W**${}_{N}^{i}$.

#### 4.3.1. Optimum Window Size

Researchers have investigated numerous aspects of window size for the CAN-bus in-vehicle network and discovered that selecting the optimal window size for intrusion detection is critical. The limitations of windowing-based methods are shown in Table 5.

In 2020, Ohira et al. [18] used the offline learning phase to determine the similarity values for various window sizes. They demonstrated that changing the sliding window size affects the similarity values. When window size *W* is set to 5, the similarity value ranges between 0.1 and 1.2, but when *W* is set to 50, the similarity ranges between 0.8 and 1.0. Furthermore, the similarity value approaches 1.0 when *W* is between 100 and 200. This method shows how similarity values increase as window sizes increase. They only tested their intrusion detection system against a DoS attack. However, to detect changes in in-vehicle traffic timing, Tomlinson et al. [19] performed a statistical analysis of CAN broadcasts. Three distinct detection methods (ARIMA, Z-score, and supervised threshold) are used to implement time-defined windows-based IDS. Each window’s metrics were calculated and then applied within that window to aid in identification. Fuzzing attacks are undetectable when preceded by other attacks because this method lacks metrics-based comparison with previous windows. Baldini [20] proposed a sliding window entropy method that employs several entropy measures during the evaluation process. This approach assesses the effects of different hyperparameters, such as window size and threshold range. However, the proposed scheme has no rationale despite extensive testing, and the results are tested on a single dataset.

This subsection investigates the effects of a change in window size when the MR is calculated against an increasing attack ratio, a topic not previously discussed. In the presence of an increasing attack ratio, we intend to provide a rationale for selecting the optimal window size. Now, it is understood that the size of the window will influence the performance of the proposed intrusion detection systems. Consequently, what is the optimal window size? This is an unanswered question since the optimal window size also depends on the other hyperparameters utilized by the proposed scheme.

#### 4.3.2. Evaluation Based on Variation in Window-Size

**Figure 7:**
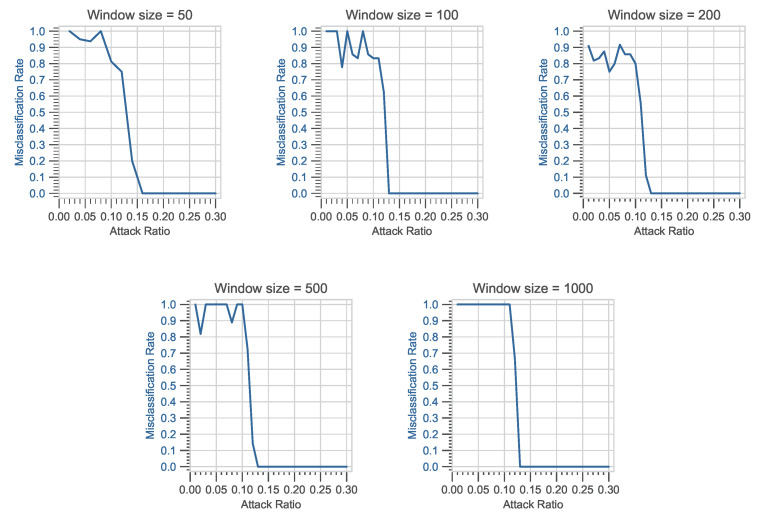
Attack ratio vs. misclassification rate for different window sizes (DoS attack).

**Figure 8:**
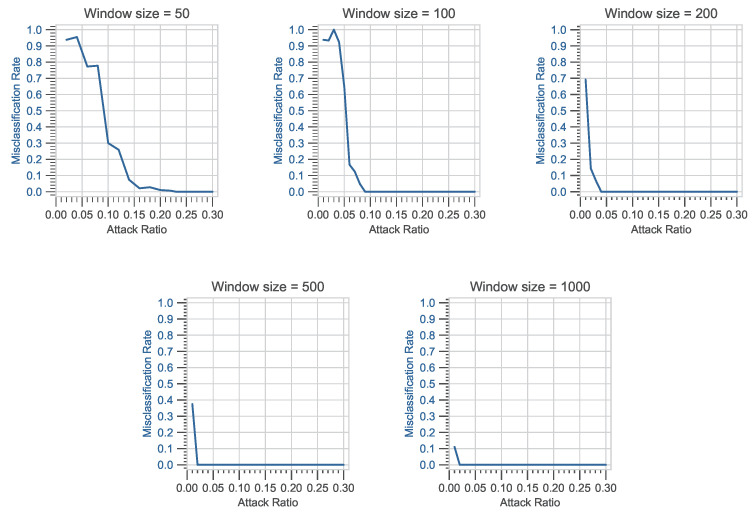
Attack ratio vs. misclassification rate for different window sizes (Fuzzy attack).

For DoS and fuzzy attack datasets, the *x*-axis represents the attack ratio, while the *y*-axis represents the MR, as shown in Figure 7 and Figure 8. The MR is calculated against the attack ratio using different window sizes. For each of the window sizes used, the MR shows a high value against a smaller value of attack ratio. Further investigation is carried out, and the attack ratio is divided into smaller blocks to fully comprehend the profound effects of window size on the MR during the attack phase. To accurately measure the MR, windows with $\epsilon^{i}$ and windows with $\epsilon^{i}$ that includes misclassification are calculated. The $\epsilon^{i}$ is broken up into smaller blocks of length 0.01, and MR is calculated by dividing the number of windows with $\epsilon^{i}$ that have misclassifications by the total number of windows with $\epsilon^{i}$. It is easily observed that the MR value is high in the beginning but goes to zero after the attack ratio goes above 0.3. Furthermore, this initial high value of MR can be observed in both attack scenarios. In the DoS attack scenario, the size of the window increases from 50 to 200, the peak value of MR slightly decreases from 1.0 to 0.9. Later, the MR rises again as the size of window increases from 200 to 1000. Moreover, for a fuzzy attack, as the size of window increases from 50 to 200, the peak value of MR remains constant, but as the window size further increases from 200 to 1000, a sharp drop in MR value is observed. This means that the size of the window is not the only thing that affects the MR value in either case.

Further investigation is carried out, and for each of the window sizes, TE is calculated to comprehend the profound effects of window size on the MR during the attack phase. The data in Table 6 indicate that TE values do not increase monotonically with increasing window size. In the DoS attack scenario, it is noticeable that as the window size increases from 50 to 100, the TE value decreases. Later, the TE value rises slightly as the window size increases from 200 to 1000. This implies that the window size does not solely influence the TE and MR values for both scenarios. As shown in Table 7, the patterns in the data for fuzzy attacks represent variations that may be inherent in the attack scenario. The TE appears to have randomly distributed values with no discernible pattern for different window sizes.

### 4.4. Threshold vs. Total Error Rate

The experimental measurements for the simulation results in this subsection were done using the following parameters. To find the minimum value of TE, different fixed-size window vectors with *N* = 50, 100, 200, 500, and 1000 were analyzed. Like in the previous experiment, *K* = 2,500,000 data frames are evaluated using the car hacking data set. The *i*-th index has a maximum value of 50,000, 25,000, 12,500, 5000, and 2500 for each value of *N*. The reference values of normality are determined using 100 legitimate window vectors for each *N* with different window size from DoS and fuzzy attack datasets, respectively. The intrusion detection function $f(\mu^{i},\sigma^{i})$ uses two thresholds, $\gamma_{1}$ and $\gamma_{2}$. The thresholds range between 1.0 and 5.0. For this experiment, each threshold is divided into 0.1 step increments for different *N* values.

In the previous section, a thorough investigation was conducted into the effects caused by the change in window size. However, in the presence of a malicious attack, it was noticed that relying solely on window size to obtain the optimal value of TE and MR is insufficient. MR is not only affected by window size but is also dependent on $\gamma_{1}$ and $\gamma_{2}$. In the following experiment, $\gamma_{1}$ and $\gamma_{2}$ values are altered against DoS and fuzzy attack datasets while different window sizes are used. The reference values of normality are calculated for each window size used. Various performance metrics are also calculated by adjusting the parameters of the intrusion detection model to obtain AER and MR.

#### 4.4.1. Limitations in Related Work

We intend to provide a rationale for selecting the best threshold value that has not yet been debated. Sagong et al. [21] investigated masquerade attacks and developed a Maximum Slackness Index metric to evaluate the efficiency of a clock skew-based IDS. They carried out the cloaking attack and demonstrated that it could avoid both IDS, specifically, the most recent state-of-the-art IDS and Network Time Protocol (NTP). Even though multiple added delays were quantified, the mechanism for threshold selection and the impact caused by variations in values of γ and Γ were not thoroughly investigated. In [22], a clock-based intrusion detection system (CIDS) used a fixed predefined threshold value, $\Gamma_{L}$ = 5, to detect anomalies. Although CIDS detects in-vehicle intrusions with a low FPR of 0.005% using cumulative sum (CUSUM) analysis, little attention has been paid to determining an optimum threshold value. Ying et al. [23] proposed a novel masquerade attack called the cloaking attack and conducted analyses of clock skew-based IDS for automotive CAN systems. Although they showed a low average prediction error, they still did not address the effect of threshold variation on MR.

#### 4.4.2. Brute Force-Based Optimization

Optimization by brute force is a straightforward method. It requires a significant amount of computing power because it evaluates all possible solutions before selecting the optimum values. This method only applies to small problems because the number of possible system states grows exponentially with the number of dimensions. For continuous predictor variables, the number of states is infinite. Despite these shortcomings, brute force methods have several advantages: they are straightforward to implement and check all possible states in a discrete system. As a result, brute force methods are frequently used to calculate the number of states or calculations required to find the optimum state. Assume this is impossible due to the presence of continuous variables. In that case, for each continuous variable, all possibilities must be tested.

#### 4.4.3. Optimization of $\gamma_{1}$ and $\gamma_{2}$

We intend to provide a reason for selecting the optimum threshold value that has not been discussed in the previous work. Table 8 shows that previous work using threshold configurations is far from having optimal values. Although, refs. [21,22,23] show improved performance, yet fail to demonstrate that MR is dependent on threshold variation.

**Figure 9:**
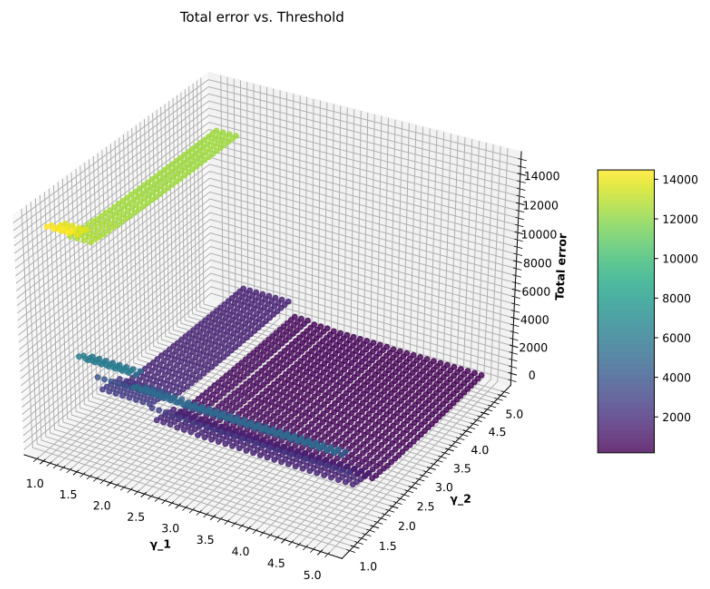
Total error vs. threshold.

As is apparent from Figure 9, when different combinations of $\gamma_{1}$ and $\gamma_{2}$ are used for a fixed window vector **W**${}_{N}^{i}$, the intrusion function $f(\mu^{i},\sigma^{i})$ produces error values that vary over a wide range. It is now recognized from Figure 9 that the threshold value significantly impacts the IDS performance. In our proposed IDS, the brute force approach is used as an optimization technique to obtain the lowest total error. The Algorithm 1 generates all possible combinations and chooses the optimum value of $\gamma_{1}$ and $\gamma_{2}$ against the applied attacks. The reference values of normality are calculated and integrated into $f(\mu^{i},\sigma^{i})$ as a part of the intrusion detection function for each window vector **W**${}_{N}^{i}$. The TE is calculated for all combinations of $\gamma_{1}$ and $\gamma_{2}$. Only one of the many possible combinations that gives the optimal value of TE, AER, and MR is chosen.
**Algorithm 1:** Minimum value of TE
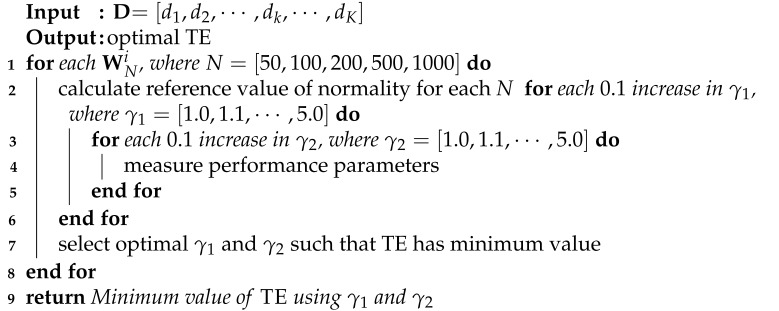


#### 4.4.4. Evaluation Based on Threshold Variation

In this subsection, we examined the proposed IDS accuracy using a variety of performance metrics. For DoS attacks, Table 9 shows the false negative error rate (FNER), false positive error rate (FPER), TE, AER, and the MR. Various window sizes are used to determine the minimized error rate using an optimal threshold value. As the window size increases, the MR increases proportionately. In the case of a fuzzy attack, Table 10 demonstrates that increasing the window size significantly reduces the MR to zero.

### 4.5. Merged Attack

Various attacks are injected into the CAN bus network in real-world scenarios. We determined the robustness of our proposed algorithm when multiple intrusions simultaneously target the CAN bus network as used in [25]. In this analysis, DoS and fuzzy attacks are merged simultaneously. A merged attack is used to create a realistic attack scenario. Two fundamental attack types are used as in combination to compromise the in-vehicle CAN bus network (i.e., DoS and fuzzy attacks). Both datasets contain exploited data frames from the car hacking dataset [26]. They created DoS and fuzzy attack datasets using the CAN bus but did not include merged attacks in their study. The following parameters were used in the experimental measurements for the simulation results in this subsection. To identify the minimum value of TE, different window vectors with size *N* = 50, 100, 200, 500, and 1000 were evaluated. A total of *K* = 3,000,000 data frames are evaluated. The reference values of normality are determined using firstly 100 legitimate window vectors for each different window of size *N*. The intrusion detection function $f(\mu^{i},\sigma^{i})$ uses two optimal thresholds values, $\gamma_{1}$ and $\gamma_{2}$. According to the data in Table 11, the window size is changed to obtain the minimized TE using optimal threshold values.

As the window size increases, the MR also increases proportionately. These results show that the behavior of the proposed IDS against a merged attack is similar to a DoS attack when injected alone. However, for the fuzzy attack, an increase in window size significantly reduced the MR to zero.

### 4.6. Comparison with Known IDS (Car Hacking Dataset)

The suggested technique is compared with recent methodologies [40,43,44,45,46], as shown in Table 12 and Table 13 against DoS and fuzzy attacks obtained from the car hacking dataset. Although most of the methods achieve high accuracy, our proposed IDS shows improved performance compared to other methodologies. The highest values for each performance criterion are bolded. Other methods have achieved a slightly higher precision or recall value in the case of a DoS attack, but the accuracy and F1 score are still lower than our proposed model. When it comes to fuzzy attacks, our IDS outperforms the rest of the proposed methods. To summarize, even though comparing existing methodologies is not an easy task, the proposed IDS outperforms them by achieving a higher score in quantitative comparison. The experimental findings indicate that the proposed IDS can effectively distinguish between legitimate and malicious data for CAN bus systems.

### 4.7. Test Case: Survival Analysis Dataset

Actual vehicle datasets were evaluated to demonstrate that the applicability of the proposed IDS is not limited to a single vehicle model. This subsection shows the performance of the proposed IDS when applied to unseen datasets from various vehicles. The test dataset is divided into normal driving data that do not involve an attack and abnormal driving data collected during an attack. The data were obtained using the Raspberry Pi31 and PiCAN22, connected to the OBD-II port via a serial peripheral interface of Sonata (2010) by HYUNDAI, Soul (2015) by KIA, and Spark (2015) by CHEVROLET. We evaluated the performance of the proposed method in terms of accuracy, precision, recall, and F1 for the survival analysis dataset [27] developed by the Hacking and Countermeasure Research Lab, Korea. The evaluation process is carried out by selecting optimal values of $\gamma_{1}$ and $\gamma_{2}$ using a brute force approach for various window sizes; Table 14, Table 15 and Table 16 show the highest accuracy of proposed IDS against DoS and fuzzy attacks.

It is validated through experimentation that the best performance can be acquired by selecting optimal threshold values for each window size. Our proposed IDS can detect DoS and fuzzy attacks with 100% accuracy against different vehicles.

#### Comparison with [47]

This subsection demonstrates how our suggested technique performed on the survival analysis dataset [27]. The comparison of our approach with [47] is shown in Table 17. It can be seen in [47] that a long short-term memory (LSTM)-based IDS is used against DoS and fuzzy attacks, respectively.

This technique thoroughly studies hyperparameter values to achieve high detection accuracy. Similar performance metrics, i.e., F1, accuracy, precision, and recall are used for evaluation purposes to conduct a fair assessment. As a result, the proposed method can become a concrete framework to identify near-real-time events with high level of accuracy. This concept will aid in the future consideration of proposed IDS for in-vehicle networks.

## 5. Limitation and Future Work

This section discusses the limitations of the proposed method and future directions for further improvement. Legitimate CAN-bus data frames must be used to establish reference values of normality to detect the intrusion. The proposed IDS detects the presence of an attacker ECU by comparing *i*-th window vector **W**${}_{N}^{i}$ to reference values of normality. However, anomaly identification may be imprecise if the compromised ECU initiates an attack prior to establishing normality values. To address this shortcoming, our IDS can obtain the normality values of those ECUs during production and update them later via software. As a result, the proposed method can be used without requiring the ECU’s hardware to be changed, but firmware must be updated.

Our method employs the sliding window method, where the size of each window remains constant throughout the analysis. In the future, we intend to update the size of each window on the fly for normal and attack scenarios to optimize performance through statistical analysis. Additionally, we intend to release novel attack models and datasets based on real-world scenarios that will serve as a sufficient challenge for researchers to develop more capable CAN-bus in-vehicle network security mechanisms to combat the latest cyber-attacks.

## 6. Conclusions

We have designed, developed, and implemented a parametric-based optimized threshold sliding window approach for intrusion detection using statistical analysis methods. The intrinsic features of the CAN bus network have been exploited to achieve a minimized MR. The performance of our proposed IDS has been evaluated using various metrics. We have investigated experimental relationships and numerical analysis for attack ratio, average, and standard deviation. We have obtained the minimized MR by optimizing the threshold values for different window sizes using brute force optimization. Additionally, we have thoroughly analyzed the merge attack as well. Two real-world datasets were evaluated to prove the usefulness of the proposed IDS for different vehicle models.

## Figures and Tables

**Table 1:** List of key notations.

Notations	Description
*N*	Window size
*K*	Total number of data frames and multiple of *N*
*k*	*k*-th data frame for 1≤k≤K
**D**	**D** be the identifier vector
$d_{k}$	Identifier of the *k*-th data frame
*Q*	Total number of identifiers in the data frames
*i*	1≤i≤K/N
*q*	1≤q≤Q
**Y** ${}^{i}$	Window’s frequency vector
$I^{i}$	Index set of identifier with non-zero frequency
$\mu^{i}$	Average of identifiers in **Y**${}^{i}$
$\sigma^{i}$	Standard deviation of identifiers in **Y**${}^{i}$
$\mu_{a}$	Average of averages
$\sigma_{a}$	Standard deviation of averages
$\mu_{s}$	Average of standard deviations
$\sigma_{s}$	Standard deviation of standard deviations
$M^{i}$	The number of attack frames in **W**${}_{N}^{i}$
$\epsilon^{i}$	The attack ratio of the *i*-th window vector **W**${}_{N}^{i}$
α	Minimum value of attack ratio in **W**${}_{N}^{i}$
$\gamma_{1}$ and $\gamma_{2}$	Threshold values

**Table 2:** Summary of the related methodologies for the CAN bus.

Proposed by	Attack Types	IDS Features	Contribution	Limitation
Cho and Shin (2016) [22]	Fabrication, Suspension, and Masquerade attack	Recursive least squares (RLS) algorithm and cumulative sum (CUSUM) algorithm	Their IDS is invulnerable to attackers who use faked timestamps	ECUs that generate aperiodic messages are challenging for the algorithm to fingerprint
Cho and Shin (2017) [33]	Impersonation attacks	It performs an online update of voltage-based fingerprints	ECU profiling based on voltage parameters to identify aperiodic attack	Voltage profiles for ECUs are required during the manufacturing stage and are updated through voltage profile adjustments
Sagong et al., (2018) [34]	Overcurrent, DoS, and forced retransmission attack	IRS uses fuses or circuit breakers	IRS provide a hardware-based system to mitigate attacks	Hardware failure requires manual replacement
Li et al., (2021) [35]	Masquerade attack	Least square estimation to build fingerprint model	Masquerade attack detection in presence of temperature variations with 2.7% false alarm rate	The detection rate of this scheme is only optimal with temperature variation
Taylor et al., (2015) [14]	Packet injection attack	Optimum window size is used	Inter-packet timing is calculated using sliding window and average times are compared to historical averages to determine anomaly	Experimental data lack non-periodic packet for anomaly detection
Song et al., (2016) [36]	Injection attacks	Entropy-based anomaly detection method	Lightweight IDS based on time intervals that detects injection attacks faster	Unable to recognize irregular message sequence and reply attacks
Müter and Asaj (2011) [37]	DoS and Spoof attack	Concept of relative entropy is used	A threshold-based detection method that reduces false-positives	Unable to identify small scale attacks
Marchetti et al., (2016) [38]	Reply and fuzzy attack	For each identifier, the algorithm tunes the model	Independent of CAN-bus messages content for anomaly detection	This method fails for IDs with high entropy variations under normal conditions.
Wu et al., (2018) [39]	DoS and injection attack	Optimum window size and threshold values are used	Heuristic algorithm based on simulated annealing for detection	A fixed size sliding window method is implemented
Seo et al., (2018) [40]	DoS, fuzzy, and RPM/Gear attacks	Generative adversarial net (GAN) is used for anomaly detection	GIDS uses a discriminator network trained on legitimate data to detect unknown attacks in changing environments	GIDS is incapable of detecting malicious or component-failure data
Longari et al., (2021) [41]	DoS, fuzzy, Replay and Sniffing	The IDS employs LSTM autoencoders	LSTM-autoencoder that uses an unsupervised learning method for detection	Complex computation for in-vehicle environment
Amato et al., (2021) [42]	DoS, fuzzy, and RPM/Gear attacks	MultiLayer perceptrons-based detection model	Human behavior-based intrusion detection	The lack of ensemble learning limits the performance of the model

**Table 3:** Performance metrics.

Performance Parameters	Description
TNW	Total number of normal windows
TAW	Total number of attacked window
FN	False negative
FNER	False negative error rate
FP	False positive
FPER	False positive error rate
TE	Total error
MR	Misclassification rate
AER	Average error rate
F1	F1-score
RCL	Recall
PRC	Precision
ACC	Accuracy

**Table 4:** Detailed description of datasets used in performance evaluations.

Datasets	Vehicle Type	Attack Types		Number of Samples		Attack Percentage	
		Total	Used	DoS Attack	Fuzzy Attack	DoS%	Fuzzy%
Car hacking dataset	Sonata	4	2	3,665,771	3,838,860	19.086	14.695
Survival analysis dataset	Sonata	3	2	149,547	135,670	21.680	13.354
	Spark	3	2	120,570	65,665	18.733	8.850
	Soul	3	2	181,901	249,990	18.219	15.925
Merged dataset	Sonata	4	2	1,500,000	1,500,000	25.225	13.263

**Table 5:** Limitations of methodologies using windowing as parameter.

Methodologies	Limitations
Ohira et al. [18]	They measured similarity across a range of sliding windows, but it was determined that the similarity-based IDS could only detect DoS attacks.
Tomlinson et al. [19]	Compared to a sliding window, this method reduces the frequency with which the window metrics must be recalculated. However, their IDS is incapable of detecting a fuzzy attack.
Baldini [20]	They classified a window as attacked if at least one malicious packet was present.
Our work	Address each of the preceding concerns.

**Table 6:** Total error (DoS attack).

Window Size	TAW	FP	FN	TE
50	20,554	287	106	393
100	10,409	95	88	183
200	5334	124	61	185
500	2299	10	79	89
1000	1261	0	99	99

**Table 7:** Total error (fuzzy attack).

Window Size	TAW	FP	FN	TE
50	22,509	364	112	476
100	11,384	61	50	111
200	5820	376	1	377
500	2472	240	0	240
1000	1349	6	0	6

**Table 8:** Limitations of methodologies using threshold as parameter.

Methodologies	Limitations
Sagong et al. [21]	The mechanism for threshold selection and the impact of variation in γ and Γ values were not thoroughly investigated.
Cho et al. [22]	A Clock-based IDS (CIDS) relied on a fixed predefined threshold value for anomaly detection, notably $\Gamma_{L}$ = 5. However, little attention has been paid to determining an optimal threshold value.
Ying et al. [23]	The effect of threshold variation on TE and ER in their IDS was not addressed.
Our work	Address each of the preceding concerns.

**Table 9:** Performance evaluation using optimum threshold value (DoS attack).

Window Size	Optimal Threshold		TNW	TAW	FPER	FNER	TE	AER	MR
	$\gamma_{1}$	$\gamma_{2}$							
50	5.0	5.0	29,446	20,554	0.0012	0.0068	178	0.0040	0.0035
100	3.0	4.3	14,591	10,409	0.0022	0.0098	136	0.0060	0.0054
200	4.2	5.0	7166	5334	0.0039	0.0142	104	0.0090	0.0082
500	1.8	2.5	2701	2299	0.0059	0.0243	72	0.0151	0.0143
1000	1.1	1.6	1239	1261	0.0064	0.0253	40	0.0158	0.0159

**Table 10:** Performance evaluation using optimum threshold value (fuzzy attack).

Window Size	Optimal Threshold		TNW	TAW	FPER	FNER	TE	AER	MR
	$\gamma_{1}$	$\gamma_{2}$							
50	3.3	4.8	27,491	22,509	0.0034	0.0046	223	0.0040	0.0044
100	3.0	3.4	13,616	11,384	0.0031	0.0037	94	0.0034	0.0037
200	4.8	5.0	6680	5820	0.0025	0.0010	24	0.0017	0.0019
500	4.9	4.9	2528	2472	0.0000	0.0000	0	0.0000	0.0000
1000	4.8	4.0	1151	1349	0.0000	0.0000	0	0.0000	0.0000

**Table 11:** Performance evaluation using optimum threshold value (merged attack).

Window Size	Optimal Threshold		TNW	TAW	FPER	FNER	TE	AER	MR
	$\gamma_{1}$	$\gamma_{2}$							
50	3.7	5.0	27,376	22,624	0.0014	0.0066	190	0.0040	0.0037
100	4.7	4.0	13,533	11,467	0.0019	0.0070	108	0.0044	0.0043
200	4.7	4.7	6618	5882	0.0030	0.0100	79	0.0065	0.0063
500	2.5	2.7	2459	2541	0.0040	0.0145	47	0.0092	0.0094
1000	4.6	2.0	1082	1418	0.0064	0.0331	54	0.0197	0.0215

**Table 12:** Performance analysis against DoS attack for known IDS (car hacking dataset).

Methods	Accuracy	Precision	Recall	F1
GIDS [40]	0.9790	0.9680	0.9960	-
KNN [43]	0.9740	-	-	0.9340
SVM [43]	0.9650	-	-	0.9330
WINDS [44]	0.9497	0.9797	0.9415	-
H-IDFS [45]	0.9728	**1.0000**	0.9620	0.9806
SAIDuCANT [46]	0.9808	0.9771	**1.0000**	0.9884
**Proposed IDS**	**0.9964**	0.9981	0.9931	**0.9956**

**Table 13:** Performance analysis against fuzzy attack for known IDS (car hacking dataset).

Methods	Accuracy	Precision	Recall	F1
GIDS [40]	0.9800	0.9730	0.9950	-
KNN [43]	0.9740	-	-	0.9340
SVM [43]	0.9650	-	-	0.9330
WINDS [44]	0.8778	0.9816	0.8339	-
H-IDFS [45]	0.9517	0.9955	0.9493	0.9718
SAIDuCANT [46]	0.8782	0.8639	0.9958	0.9252
**Proposed IDS**	**1.0000**	**1.0000**	**1.0000**	**1.0000**

**Table 14:** Performance parameters for Sonata.

Attack Type	Window Size	Optimal Threshold		Accuracy	Precision	Recall	F1
		$\gamma_{1}$	$\gamma_{2}$				
DoS	50	4.7	3.5	0.9993	1.0000	0.9986	0.9993
	100	2.5	3.4	0.9979	1.0000	0.9958	0.9979
	200	3.0	3.0	0.9959	1.0000	0.9917	0.9958
	500	2.6	3.6	0.9966	1.0000	0.9932	0.9966
	1000	1.8	1.4	1.0000	1.0000	1.0000	1.0000
fuzzy	50	2.6	4.1	0.9996	1.0000	0.9990	0.9995
	100	2.3	3.3	1.0000	1.0000	1.0000	1.0000
	200	3.0	3.0	1.0000	1.0000	1.0000	1.0000
	500	2.7	3.0	1.0000	1.0000	1.0000	1.0000
	1000	3.7	4.1	1.0000	1.0000	1.0000	1.0000

**Table 15:** Performance parameters for Soul.

Attack Type	Window Size	Optimal Threshold		Accuracy	Precision	Recall	F1
		$\gamma_{1}$	$\gamma_{2}$				
DoS	50	2.1	3.7	0.9991	1.0000	0.9980	0.9990
	100	2.3	2.9	0.9983	1.0000	0.9960	0.9980
	200	3.6	4.3	1.0000	1.0000	1.0000	1.0000
	500	2.2	2.0	0.9972	1.0000	0.9935	0.9967
	1000	1.8	2.8	1.0000	1.0000	1.0000	1.0000
fuzzy	50	3.3	4.2	0.9985	0.9987	0.9983	0.9985
	100	3.1	4.6	1.0000	1.0000	1.0000	1.0000
	200	3.9	4.6	1.0000	1.0000	1.0000	1.0000
	500	3.0	4.2	1.0000	1.0000	1.0000	1.0000
	1000	3.9	4.1	0.9959	0.9920	1.0000	0.9959

**Table 16:** Performance parameters for Spark.

Attack Type	Window Size	Optimal Threshold		Accuracy	Precision	Recall	F1
		$\gamma_{1}$	$\gamma_{2}$				
DoS	50	4.7	4.3	0.9995	1.0000	0.9991	0.9995
	100	3.8	4.0	1.0000	1.0000	1.0000	1.0000
	200	2.8	3.4	1.0000	1.0000	1.0000	1.0000
	500	3.1	3.3	1.0000	1.0000	1.0000	1.0000
	1000	2.7	3.3	0.9916	1.0000	0.9833	0.9915
fuzzy	50	2.6	4.1	0.9996	1.0000	0.9990	0.9995
	100	2.3	3.3	1.0000	1.0000	1.0000	1.0000
	200	3.0	3.0	1.0000	1.0000	1.0000	1.0000
	500	2.7	3.0	1.0000	1.0000	1.0000	1.0000
	1000	3.7	4.1	1.0000	1.0000	1.0000	1.0000

**Table 17:** Performance analysis against known IDS (survival analysis dataset).

Model	Attack Type	Method	Accuracy	Precision	Recall	F1
Sonata	DoS	Hossain et al. [47]	1.0000	-	1.0000	1.0000
		Proposed IDS	1.0000	1.0000	1.0000	1.0000
	fuzzy	Hossain et al. [47]	0.9996	-	1.0000	0.9999
		Proposed IDS	1.0000	1.0000	1.0000	1.0000
Soul	DoS	Hossain et al. [47]	1.0000	-	1.0000	1.0000
		Proposed IDS	1.0000	1.0000	1.0000	1.0000
	fuzzy	Hossain et al. [47]	0.9962	-	0.9763	0.9880
		Proposed IDS	1.0000	1.0000	1.0000	1.0000
Spark	DoS	Hossain et al. [47]	1.0000	-	1.0000	1.0000
		Proposed IDS	1.0000	1.0000	1.0000	1.0000
	fuzzy	Hossain et al. [47]	0.9960	-	0.9780	0.9780
		Proposed IDS	1.0000	1.0000	1.0000	1.0000

## Data Availability

Not applicable.
